# Functional Roles of RNA-Binding Proteins in Plant Signaling

**DOI:** 10.3390/life10110288

**Published:** 2020-11-18

**Authors:** Victor Muleya, Claudius Marondedze

**Affiliations:** 1 Department of Biochemistry, Faculty of Medicine, Midlands State University, Main Campus, Senga Road, Gweru P Bag 9055, Zimbabwe; muleyav@staff.msu.ac.zw; 2 Rijk Zwaan, 2678 ZG De Lier, The Netherlands

**Keywords:** RNA-binding protein, RNA interactome capture, signaling, abiotic stress, mass spectrometry

## Abstract

RNA-binding proteins (RBPs) are typical proteins that bind RNA through single or multiple RNA-binding domains (RBDs). These proteins have a functional role in determining the fate or function of the bound RNAs. A few hundred RBPs were known through in silico prediction based on computational assignment informed by structural similarity and the presence of classical RBDs. However, RBPs lacking such conventional RBDs were omitted. Owing to the recent mRNA interactome capture technology based on UV-crosslinking and fixing proteins to their mRNA targets followed by affinity capture purification and identification of RBPs by tandem mass spectrometry, several hundreds of RBPs have recently been discovered. These proteome-wide studies have colossally increased the number of proteins implicated in RNA binding and unearthed hundreds of novel RBPs lacking classical RBDs, such as proteins involved in intermediary metabolism. These discoveries provide wide insights into the post-transcriptional gene regulation players and their role in plant signaling, such as environmental stress conditions. In this review, novel discoveries of RBPs are explored, particularly on the evolving knowledge of their role in stress responses. The molecular functions of these RBPs, particularly focusing on those that do not have classical RBDs, are also elucidated at the systems level.

## 1. Introduction

RNA-binding proteins (RBPs) are ubiquitous in living systems from unicellular to multicellular organisms. Apart from their apparent role in determining the fate of RNA molecules, they play crucial roles in a myriad of cellular processes including the regulation of gene expression in response to different environmental stimuli [1]. These RBPs permit cells to rapidly alter their expression patterns in response to various environmental stimuli. A quick response is particularly detrimental in unicellular organisms that are largely dependent on their ability to acclimatize to environmental changes and to survive. 

RNA interacting proteins have been reported in unicellular organisms, including the unicellular alga *Chlamydomonas reinhardtii* [2], in the protozoan parasitic euglenoid *Trypanosoma cruzi* [3], and several bacterial species, including *Shigella sonnei* [4], *Enterococcus faecalis* [5] and *Bacillus subtilis* [6]. Surprisingly, most of the identified bacterial RBPs seem to lack identifiable RBDs and are equipped with metabolic enzymatic function, allowing them to moonlight as RBPs [7,8]. Despite the lack of compartmentalization, some of the RBPs identified in unicellular organisms have been shown to play a crucial role in RNA cellular localization [4,9]. A new bacterial RBP with membrane localization was shown to confer tolerance to alcohol- and cold-induced stress in certain fermicutes [10]. Taken together, these observations accentuate the ubiquitous occurrence of RBPs in living systems and further highlight their moonlighting functions found in unicellular organisms.

RBPs have also been identified in both yeast and mammalian cell extracts. In humans, more than a combined thousand proteins in the human embryonic kidney (HEK-293) cells were identified to have RNA binding properties [11,12]. As expected, a large representation of these mammalian proteins had canonical RNA-binding domain (RBD) sequence signatures and had previously been functionally annotated as RBPs. Surprisingly, a significant portion of these summative candidate RBPs, were found to be either devoid of any structural homology to known RBPs, or previously illustrated RNA-binding activity [11]. In the yeast *Saccharomyces cerevisiae*, close to 700 RBPs have been identified [13], of which 60% have previously assigned functions in RNA biology. These proteins shared common structural motifs that are known to confer RNA binding activity, with a sizeable proportion of these consisting of a majority of enzymes found in the glycolytic pathway. The striking identification of enzymatic RBPs is indicative of a strong association between gene regulation and metabolism.

RBPs are functionally conserved in *Arabidopsis thaliana* and *Oryza sativa* during the cold stress adaptation process [14]. In Arabidopsis, four major studies cumulatively identified 2701 putative RBPs (reviewed in [15]) in UV-crosslinked fractions of leaves and cell suspensions [16,17], etiolated seedlings [18], leaf mesophyll protoplasts [19], and leaves [20]. These exploratory undertakings unearthed a number of plant proteins previously not assigned to RNA biology. Most of the noncanonical RBPs have been shown to play crucial roles in stress signaling, with some of them being involved in the mainstream of carbohydrate metabolic pathways like glycolysis and the tri-carboxylic acid cycle [16]. This observation suggests that these previously unassigned RBPs may play crucial functions in regulating metabolic changes in response to various stress stimuli.

This review will address some of the pertinent novel discoveries of RBPs in plants and their role in stress response. The molecular functions of these RBPs that do not have classical RBDs will also be elucidated.

## 2. High-Resolution Determination of RNA-Binding Proteins

There are several techniques, which have been used in the identification of RBPs, each with its own advantages and limitations. Most notably, several homology-based in silico prediction techniques that employ computational assignment strategies that heavily rely on detecting structural similarities and the presence of classical RB domains have unearthed a few hundred RBPs [21]. Most recently, a homology-based technique that uses machine learning, with only the protein sequence as input, has been used to uncover nucleic acid-binding proteins, including RBPs [22]. However, these methods have inborn caveats, such as providing a limited proteome coverage and the occurrence of high false positives. For instance, RBPs that are devoid of classical RBDs will be omitted entirely, thereby conferring limited proteome coverage to these in silico techniques. 

Due to the glaring limitations of in silico RBP identification techniques, high-resolution proteome-wide studies have closed the gap and led to an increase in the identification of candidate proteins implicated in RNA binding. For instance, genome-wide protoarrays and fluorescent RNA probes have previously been used in the identification of RBPs in yeast [23,24]. In some studies, an MS-based technique that employs aptamer-tagged RNA as bait has been used to capture RBPs [25]. Most interestingly, the fairly recent mRNA interactome capture (RIC) technology, which has been optimized for plants, furnishes the best example. This technology has unraveled several hundreds of novel RBPs lacking classical RBDs, such as proteins involved in intermediary metabolism [17]. The RIC technology is based on UV-crosslinking and fixing proteins to their putative mRNA targets. Following purification by affinity capture, candidate RBPs are then identified using tandem mass spectrometry. The strategy yielded well over a thousand different proteins together with several hundreds of proteins that were functionally classified as RNA-binding with known RBDs or harbored orthologs identified in mammals, *C. elegans*, or *S. cerevisiae*. In addition to these classical RBPs, the RIC technology unearthed over 1800 novel candidate RBPs that are devoid of typical RBDs, thereby broadening our understanding of the RBP repertoire in general [15].

Despite its celebrated success in the discovery of putative RBPs, the presence of ribosomal RNA and DNA contamination is a consistent limitation that is associated with RIC [26]. A modified RIC protocol, known as enhanced RIC (eRIC), which tries to minimize the caveats associated with RIC, has recently been established. This technique employs the use of a locked nucleic acid (LNA)-modified capture probe, which purportedly offers greater specificity and heightened signal-to-noise ratios relative to unmodified RIC [27]. Due to the increased signal-to-noise ratios associated with eRIC, this technique allows for the detection of more RNA-protein interactions that would otherwise evade the analysis of unmodified RIC. For instance, one study noted that in cells treated with a potent RNA demethylase inhibitor, eRIC detected m6A-responsive RBPs that evade RIC detection [27]. Besides, the benefits of this technique and other recent modified versions of RIC are yet to be gained in plant systems. 

## 3. Dual Functional Networks of RNA-Binding Proteins

Various proteins involved in intermediary metabolism or rather enzymes of intermediary metabolic process were detected in mRNA interactomes. Catalogues of mRNA-bound proteins now suggest a more general functional relevance of enzymes moonlighting as RBPs supporting an earlier RNA, enzyme, and metabolism (REM) hypothesis that proposes a link between metabolism and RNA-based regulation of gene expression [28]. Notably, in addition to proteins controlling the fate of bound mRNA, the RNAs could in turn, serve as regulators of enzymatic activity, possibly through competition or allosteric activation/repression, or by acting as scaffold for the assembly of enzyme complexes [29].

Comparatively, the Arabidopsis, mammalian, *C. elegans* and *S. cerevisiae* systems, uncovered a common set of enzymes that have a role in intermediate metabolism, including enzymes involved in the glycolysis and tri-carboxylic acid cycles [17]. This inter-specific comparative analysis also revealed some distinct differences in the RBP repertoires of each organism, suggesting that these RBPs are also tissue and species-specific. Interestingly, some of these enzymes of the intermediary metabolic pathway were modified under drought stress conditions, indicative of a link between post-transcriptional gene regulation and stress-induced metabolic changes either via RBPs regulating their own mRNAs or vice versa [16]. In response to drought stress, four carbohydrate metabolism enzymes, glyceraldehyde 3-phosphate dehydrogenase C-2 (GAPDH), aldehyde dehydrogenase 7B4, pyruvate dehydrogenase E1 component and aconitase, which are also responsive to abscisic acid (ABA) stimulus and water deprivation, are among the enzymes that were detected as differentially regulated at their RNA interaction levels. For example, at the protein level, the expression of glyceraldehyde 3-phosphate dehydrogenase increases in response to cold stress [30] and in response to drought stress, an increase was noted at post-transcriptional level, denoting a potential transcriptional rise of its target RNA. 

Increasing evidence on the enzyme-RNA interaction sheds light on the role of metabolic enzymes in dual functionality. For example, in non-plant systems, in vivo and in vitro evidence confirm the existence of RNA-binding activities within the nicotinamide adenine dinucleotide (NAD)-binding pocket of GAPDH [31,32]. Just like in the mammalian system, eight GAPDH NAD-binding and GAPDH C-terminal domain-containing proteins were observed in plants [15]. GAPDH has been shown to bind to diverse RNA species, including AU-rich elements, tRNAs and telomerase RNA component (TERC) [33,34]. Based on evidence from other systems, it is imperative to suggest that the same principle is conserved in plants, and that GAPDH potentially interact with RNA through its NAD-binding pocket. 

## 4. RNA-Binding Proteins Modulate Stress Response Signals 

In higher organisms, including plants, direct or indirect binding of RBPs to target RNAs plays a crucial role both under normal and traumatic cellular conditions. Indirect binding is generally achieved through the interaction of RBPs with each other in their regulatory roles such as in the formation of stress granules during stress responses. There has been a paucity of information on the role of plant RBPs in stress, relative to other organisms like, yeast, bacteria and humans. However, relatively recent and extensive studies have managed to cover the gap in our understanding of the emerging roles of plant RBPs in stress response and signaling (reviewed in [1,35,36]).

The association of proteins with mRNAs is very dynamic and prone to changes according to the environment. Accordingly, traumatic stress or extracellular signals elicit an RBP-dependent stabilization or destabilization of mRNAs [37]. In plants, this is best demonstrated by the crucial role that RBPs play in thermotolerance during heat shock stress. For instance, heat shock protein 101 (HSP101), an RBP from Arabidopsis, has been shown to chaperone the release of ribosomal mRNAs from stress granules after heat shock [38]. Furthermore, by using ^15^N-labelling, the authors were able to show that new ribosomes can be produced in a transcription-independent manner, consequently signaling the resumption of translation during the recovery period. The observed production of new ribosomal proteins could be a mean of rapidly restoring the translation apparatus post-heat shock trauma.

**Figure 1:**
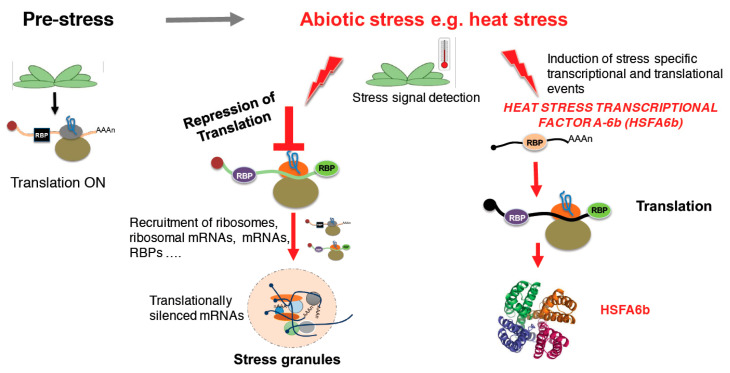
A schematic representation of stress granules formation. Under normal growth conditions, translation is ON (left panel). However, when a stress signal is detected, heat stress translation is stalled leading to the recruitment of protein-RNA complexes forming cytoplasmic foci termed stress granules [41]. On the other hand, stress induces transcription and translation of stress responsive genes and proteins, respectively, like the heat stress transcriptional factor A-6b (HSFA6b) [42], suggesting that some translation machineries are kept running to allow plants to sustain the stress and survive under such conditions.

Furthermore, HSP101 and other heat shock proteins have been implicated in the aggregation of protein translation factors and the sequestration of mRNAs into stress granules during heat shock stress [39]. The subsequent dissociation of these stress granules during the recovery phase also involves heat shock proteins. Apart from heat shock stress, in Arabidopsis, the aggregation of proteins into stress granules (Figure 1) and their subsequent dissociation have been reported to occur during hypoxic stress and ensuing reoxygenation [40].

In another study, Arabidopsis plants, overexpressing the RBP Oligouridylate Binding Protein 1b (UBP1b), displayed an increased heat tolerance relative to control plants [43]. In addition, UBP1b deficient plants were observed to be more sensitive to heat stress than control plants. Heat treatment of UBP1b-overexpressing Arabidopsis plants resulted in the formation of cytoplasmic foci, demonstrating that UBP1b is an integral component of stress granules. Using microarray analysis, the authors noted that 117 genes, which were heat-inducible, showed higher expression profiles in UBP1b overexpressing lines. In addition, the rate of RNA decay of a heat shock protein (DnaJ) and a stress-associated protein (AtSAP3) in UBP1b overexpressing plants was slower than in control plants. This additional observation suggests that the mRNAs of these heat-inducible genes were sheltered within the UBP1b stress granule during heat shock stress. Taken together, these findings suggest that UBP1b has RNA chaperone activity and confers thermotolerance to plants during heat shock stress. Elsewhere, zinc-finger-containing RBPs that have stress responsive roles have also been elucidated in *Brassica rapa* [44]. These zinc-finger-containing RBPs were shown to possess RNA chaperone activity under abiotic stress conditions as well as in the presence of ABA. To further highlight the role of RBPs in stress signaling, the gene expression of an Arginine Glycine Glycine (RGG) box-containing RBP from *Arabidopsis thaliana* (AtRGGA) was upregulated in seedlings after long-term exposure to ABA, while treatments with NaCl resulted in AtRGGA downregulation [45]. Collectively, this indicates that AtRGGA has a regulatory role during salt and drought stress tolerance. 

RBPs have been implicated in conferring cold tolerance in several plant species. For instance, the expression of the *Cucumis sativus* glycine-rich RBP (CsGR-RBP)3, was shown to be significantly upregulated under low temperatures in the cucumber fruit [46]. In the same study, a transient expression of mitochondrial CsGR-RBP3 was observed, suggesting a role for CsGR-RBP3 in maintaining mitochondria-related functions under low temperature. Furthermore, Arabidopsis mutant lines overexpressing CsGR-RBP3 showed higher survival rates at −20 °C, compared to wild-type plants. These mutant lines exhibited lower reactive oxygen species levels, and higher catalase and superoxide dismutase expression and activities, compared with the wild-type plants. Taken together, these observations indicate that the plant RBP CsGR-RBP3 performs a significant role in the adaptation of plants to cold stress.

Using gene ontology analysis, a recent study has unearthed a significant number of stress-responsive RBPs including some proteins previously not classified as RBPs [16]. Surprisingly, these putative RBPs had well-established functions in the mainstream of carbohydrate metabolism. Using the model plant Arabidopsis, the authors used label-free mass spectrometry to identify about 600 proteins, with 150 of these being sensitive to drought-induced treatment. Collectively, these findings may suggest that plant RBPs may have poorly understood functions in cellular processes that regulate metabolic changes during stress response. In a more recent study using *Arabidopsis thaliana*, changes in the components of the spliceosome were analysed during drought stress by examining alterations in the RBPs. The composition of drought-induced stress granules was also examined [15]. This study identified 12 proteins in the stress granules that were co-expressed with spliceosome proteins. These 12 proteins were identified as classical components of stress granules and their abundance was noted to increase during drought stress indicating a drought stress-induced translational arrest. Seven of these were detected to interact with mRNA after drought stress treatment. Further analysis of the entire drought stress-responsive mRNA binding proteome data revealed an overall 32 stress granule associated proteins. The observed overlap of proteins co-expressed in spliceosome assembly and stress granule formation (Figure 1), suggests some intermolecular crosstalk between the two molecular processes and potentially an important stress-induced signal modulating post-translational gene regulation during stress.

There has been a dearth of information on the role of plant RBPs during mechanical stress; however, very few studies have reported the role of plant RBPs in wounding after pathogen attack [47,48]. In one study, mRNA expression levels of a glycine-rich RBP in cultivated tobacco were measured after leaf wounding by scraping with pins and observed a 2.5-fold increase 12 h after treatment [49]. Similarly, two glycine-rich RBPs from tobacco, *Nicotiana tabacum* (Nt)GRP-1α and NtGRP-3 genes, were rapidly upregulated at 1 h post-wounding, then their expression increased until reaching a peak after 4 h of stress [50]. There is a need to further elucidate the role of plant RBPs in mechanical stress response as their mechanism of action in this role is poorly understood.

## 5. Perspectives

This review reports on the overwhelming evidence that several plant RBPs play a role in signaling various environmental stimuli. Notwithstanding the fact that current understanding of the stress responsive roles played by plant RBPs is not as advanced as that of other species, like humans. This provides the impetus to conduct more studies that aim to close the gap in our understanding. It is nevertheless noteworthy that the use of new and/or modified techniques like RIC has greatly expanded our knowledge of plant RBPs as they unearthed noncanonical RBPs involved in plant signaling. With that noted, it remains unclear how RBPs enable RNA recruitment during dehydration stress-induced during seed maturation and, furthermore, the role of RBPs in the release of these stored RNAs during release of dormancy and initiation of germination. New insights in this area will greatly advance some molecular signaling insights in seed development, storage (in particular control of dormancy) and germination. Not only will it be a key in manipulating seeds to germinate under unfavourable conditions, but it will advance food production in regions demarcated unsuitable for crop production. Besides, another important aspect that is scarcely explored in plants is understanding and uncovering the turnover and translation regulatory RBPs in general and in particular under stress conditions. In sum, the information that is currently available will act as an indispensable tool in the biotechnological engineering of plant cultivars that are more stress-tolerant.
